# Mitofusion is required for MOTS‐c induced GLUT4 translocation

**DOI:** 10.1038/s41598-021-93735-2

**Published:** 2021-07-12

**Authors:** Khushwant S. Bhullar, Nan Shang, Evan Kerek, Kaiyu Wu, Jianping Wu

**Affiliations:** 1 Department of Agricultural, Food, and Nutritional Science, University of Alberta, Edmonton, AB Canada; 2 Department of Pharmacology, University of Alberta, Edmonton, AB Canada

**Keywords:** Cell signalling, Endocrine system and metabolic diseases

## Abstract

MOTS‐c (mitochondrial ORF of the twelve S-c) is a 16-amino-acid mitochondrial peptide that has been shown to counter insulin resistance and alleviate obesity in vivo. However, the mechanisms involved in the pharmacological action of MOTS-c remain elusive. Based on the ability of MOTS-c to improve insulin resistance and promote cold adaptation, we hypothesized that MOTS-c might play a role in boosting the number of mitochondria in a cell. We found that treatment of mammalian cells with MOTS‐c increased protein levels of TFAM, COX4, and NRF1, which are markers for mitochondrial biogenesis. However, flow cytometry analysis using MitoTracker Green revealed a sharp reduction in the mitochondrial count after MOTS‐c treatment. We then anticipated possible synchronized activation of mitofusion/mitochondrial fusion by MOTS‐c following the onset of mitochondrial biogenesis. This was confirmed after a significant increase in protein levels two GTPases, OPA1, and MFN2, both vital for the fusion of mammalian mitochondria. Finally, we found that inhibition of the two GTPases by TNFα abrogated the ability of MOTS‐c to prompt GLUT4 translocation and glucose uptake. Similar results were obtained by siRNA KD of MFN2 as well. Our results reveal for the first time a pathway that links mitofusion to MOTS-c-induced GLUT4 translocation.

## Introduction

Mitochondria are essential semiautonomous organelles that play key roles in a plethora of physiological and biochemical processes^1,2^. Mitochondria coordinate intricate physiological tasks such as metabolism, oxidative phosphorylation, and stress response via interaction with nuclear-encoded factors and retrograde signaling molecules like free radicals and cytochrome *c*^1,3^. Apart from metabolism and energy production, mitochondria also relay information via mitochondrial-derived peptides (MDPs), such as humanin and mitochondrial ORF of the twelve S-c (MOTS-c)^4,5^. These MDPs have revealed a larger mitochondrial genetic repertoire and switched the perception of mitochondria from “end-function” organelles to versatile retrograde signaling organelles^1,2,6^. Amongst currently known MDPs, MOTS-c appears to mediate numerous physiological functions^7^.

Originally, mitochondrial transcriptome analyses uncovered the presence of small RNAs derived from mtDNA, culminating in the discovery of MDPs^8^. MOTS-c, a 16-amino acid peptide is encoded from the 12S rRNA region of the mitochondrial DNA. It has been shown to have the ability to regulate insulin action and cellular metabolism in an AMPK-reliant manner^3,9^. MOTS-c is expressed in muscles and other organs and is detected in plasma as well^3,9,10^. On the metabolic front, MOTS-c treatment raises NAD^+^ levels and counteracts diet-induced and age-dependent insulin resistance in mice, suggesting it has an endocrine and exercise mimetic effect^3,7^. It also counters deleterious metabolic effects related to menopause in an ovariectomized mouse model in an AMPK-dependent mannner^10,11^. Likewise, the antinociceptive effects of MOTS-c in in vivo neuropathic pain models and on osteoclastogenesis are AMPK-dependent^12,13^. Clinical findings in humans also relate lower levels of plasma MOTS-c to insulin resistance and impaired coronary endothelial function^14–16^. Also, genetic polymorphism is proposed to contribute to the risk of Type 2 diabetes (T2D) in sedentary men by diminishing MOTS-c activity via an amino acid change from *WT-*MOTS-c (K14) to *K14Q*-MOTS-c (Q14)^17^. These diverse metabolic phenotypes suggest that mitochondria may well modulate metabolism and insulin resistance in part through the synthesis of mitochondrial peptides like MOTS-c. The detailed known mechanisms underlying metabolic signaling and two‐way genomic dialogue by MOTS-c are described elsewhere^18^. Considering this important pharmacological spectrum of MOTS-c activity, we decided to pursue studies to gain understanding about its mechanistic functioning.

Despite numerous studies on the metabolic activity of MOTS-c, several gaps, such as how MOTS-c and mitochondrial mechanisms are related to glucose uptake, remain vague. A full dissection of how MOTS-c navigates insulin uptake might be related to mitochondrial retrograde signaling and the insulin-responsive glucose transporter type 4 (GLUT4). GLUT4 during its constitutively “on” phase in adipocytes and muscle cells facilitates glucose transport in response to insulin stimulation^19^. It is now well understood that mitochondria putatively control metabolism and insulin activity, in part, through GLUT4^20,21^. Interestingly, increased expression of mitochondrial fusion via modulation of MFN2 and microRNA-106b triggers the GLUT4 translocation signaling and maintenance of glucose homeostasis in vivo^22–24^. Therefore, we hypothesized that a direct connection between the two, i.e., MOTS-c and GLUT4, is likely. Further, MOTS-c treatment increases the adipose thermogenesis^25^, a process related to PGC1α activation^26^, which is a dual modulator of mitochondrial physiology and glucose uptake. Therefore, a possible relationship between MOTS-c to GLUT4 mediated glucose uptake via mitochondrial mechanisms cannot be ignored. Based on the results in this study, we propose a model relating the metabolic boosting by MOTS-c to GLUT4 translocation through activation of mitochondrial biogenesis, or a closely related mechanism leading to generation of specialized mitochondria.

## Results

### MOTS-c treatment increases mRNA levels of genes related to mitochondrial biogenesis

**Figure 1:**
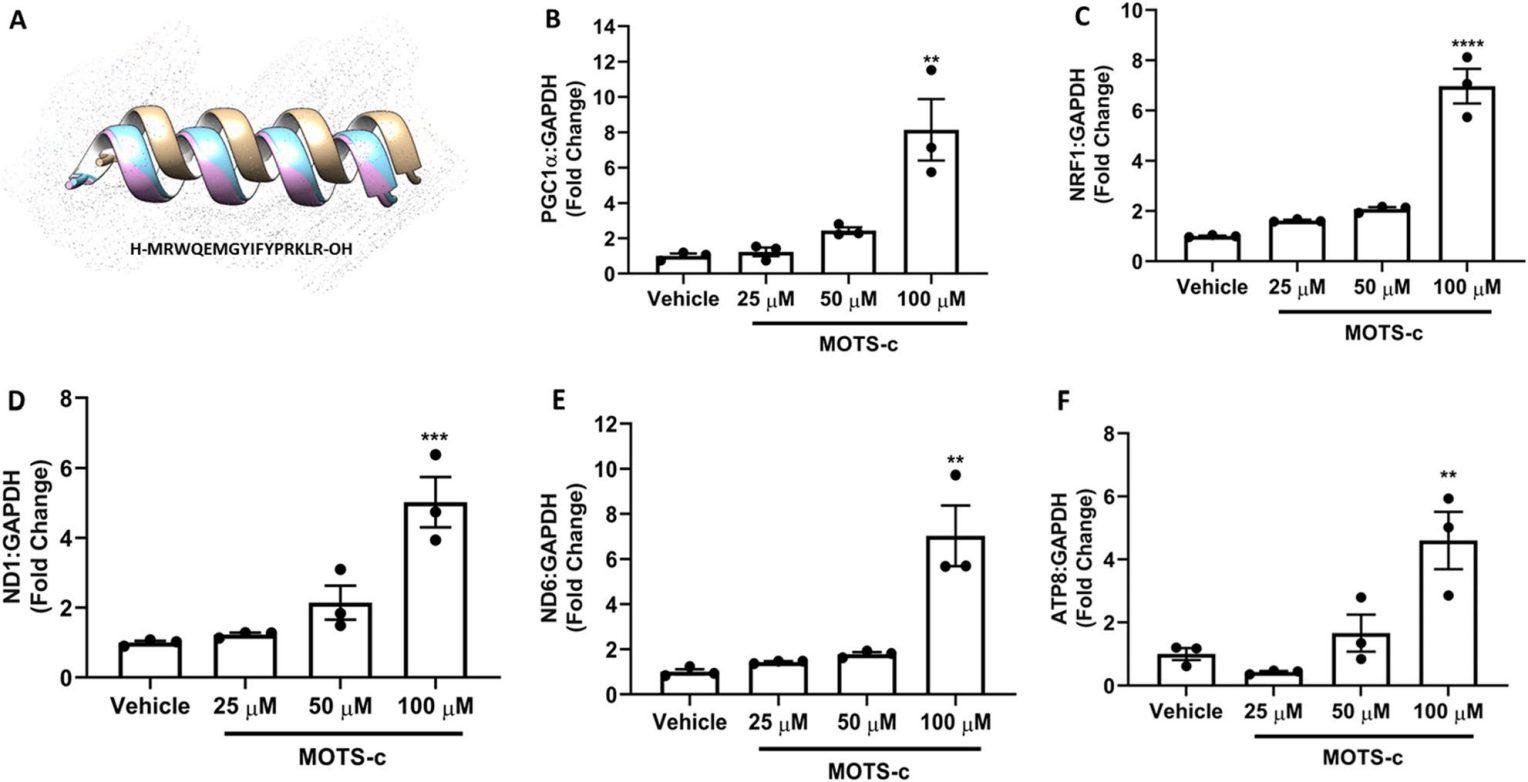
Treatment of U-2 OS cells with MOTS-c upregulated protein expression of selected biomarkers of mitochondrial biogenesis. (**A**) MOTS-c treatment increased mRNA expression of (**B**) PGC1α (**C**) NRF1 (**D**) ND1 (**E**) ND6 and (**F**) ATP8. U-2 OS cells grown in Dulbecco’s modified Eagle’s medium (DMEM) supplemented with 10% fetal bovine serum (FBS) plus 1% pen-strep were treated with vehicle (Nuclease-Free Water) or MOTS-c (25, 50 or 100 μM) for 48 h and qPCR was performed to quantify the mRNA expression levels of selected biomarkers. GAPDH was used as the internal control. Data are means ± SEM of three experiments. Statistical analysis was conducted using ordinary one-way ANOVA followed by Dunnett's multiple comparisons test vs vehicle; results are presented as *p < 0.05, **p < 0.01, ***p < 0.001 and ****p < 0.0001 vs. vehicle.

To investigate whether MOTS-c treatment can initiate transcription of genes involved in mitochondrial biogenesis, we measured mRNA levels of vital transcription factors, like PGC1α and NRF1 in U-2 OS cells. In this study, we also focused on mitochondrial gene upregulation to measure a possible increase in mitochondrial DNA (mtDNA). We found that MOTS-c treatment (100 μM) upregulated the mRNA expression of PGC1α (peroxisome proliferator-activated receptor gamma coactivator 1α) and NRF1 (Nuclear Respiratory Factor 1) by greater than fivefolds in U-2 OS cells in vitro (Fig. 1B,C). Given the extent of the mRNA upregulation of transcription factors related to mitochondrial biogenesis by MOTS-c, we expected an increase in mitochondria genes as well. Hence, we also compared the relative RNA amount of the transcripts of two mitochondrial genes, ND1 (NADH:Ubiquinone Oxidoreductase Core Subunit 1) and ND6 (NADH:Ubiquinone Oxidoreductase Core Subunit 6). A significant increase in mRNA levels of ND1, ND6 and ATP8 (p < 0.001; p < 0.01; p < 0.01) was observed with greater than fivefold enrichment (Fig. 1D–F). This increase was analogous to the increase in PGC1α and NRF1 mRNA levels; however, the lower concentration(s) of MOTS-c (25 and 50 μM) were unable to increase the mRNA levels of the investigated targets. Overall, our qPCR results show the activation of vital factors for mitochondrial biogenesis along with ensuing gene expression of ND1, ND6 and ATP8 by MOTS-c treatment (100 μM) in U-2 OS cells.

### MOTS-c treatment increases protein expression of mitochondrial biogenesis

**Figure 2:**
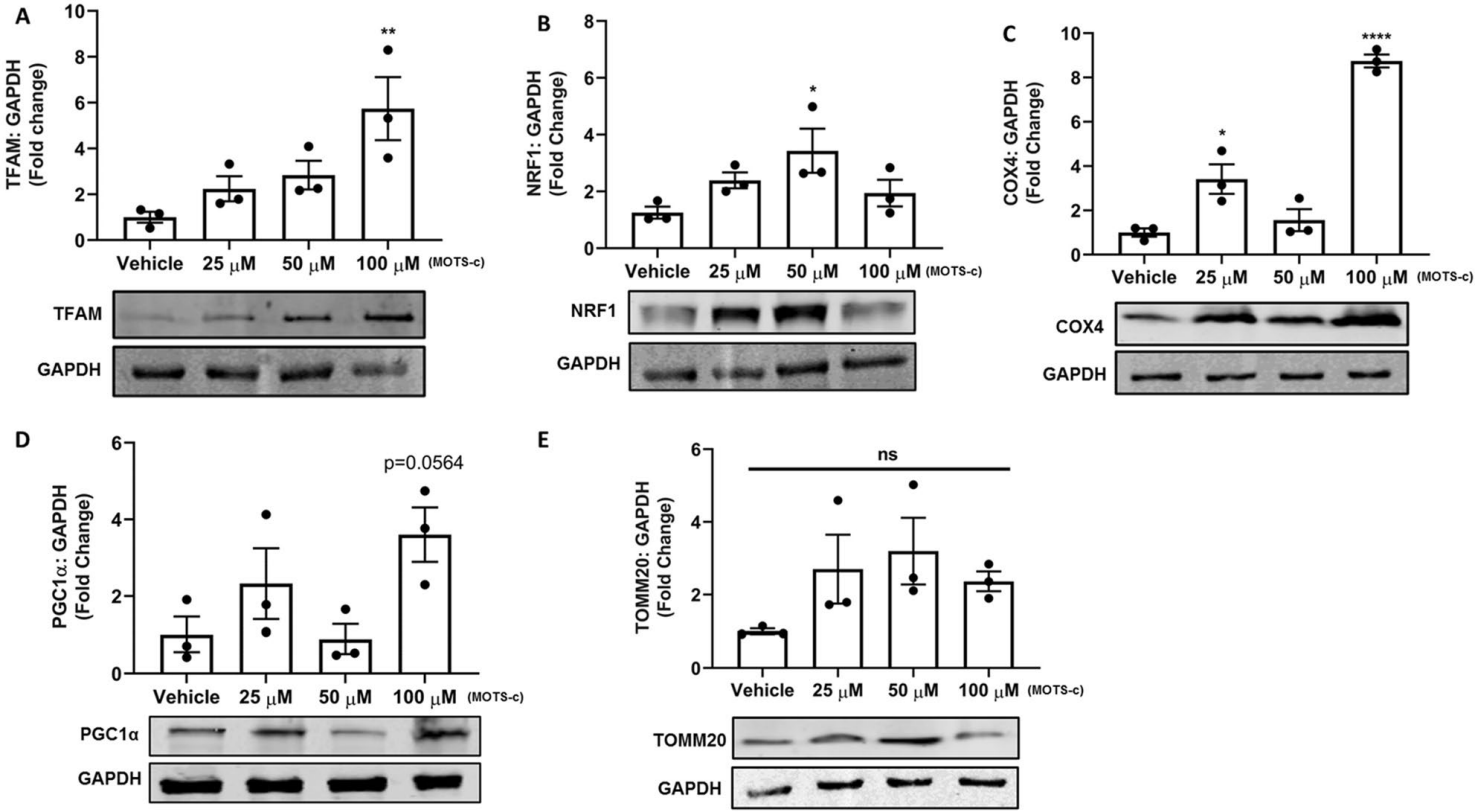
Treatment of U-2 OS cells with MOTS-c increased protein expression of (**A**) TFAM (**B**) NRF1 (**C**) COX4 but had no significant impact on (**D**) PGC1α and (**E**) TOMM20. U-2 OS cells grown in Dulbecco’s modified Eagle’s medium (DMEM) supplemented with 10% fetal bovine serum (FBS) plus 1% pen-strep were treated with vehicle (Nuclease-Free Water) or MOTS-c (25, 50 or 100 μM) for 48 h and western blot was performed to quantify the protein expression level of selected biomarkers. GAPDH was used as the internal control. All full-length blots are presented in Supplementary Figure WB1. Data are means ± SEM of three experiments. Statistical analysis was conducted using ordinary one-way ANOVA followed by Dunnett's multiple comparisons test vs vehicle; results are presented as *p < 0.05, **p < 0.01 and ****p < 0.0001 vs. vehicle.

To further explore and confirm the activation of mitochondrial biogenesis, the downstream effectors of the PGC1α pathway were evaluated by immunoblotting. We identified that MOTS-c treatment had a significant positive impact on protein expression of TFAM, COX4, and NRF1 in U-2 OS cells. The protein levels of TFAM, a key enhancer of mitochondrial biogenesis^27^, and NRF1, which regulates expression of TFAM^27^, were significantly increased (p < 0.01; p < 0.05) by MOTS-c treatment (100 μM) in U-2 OS cells (Fig. 2A,B). Similarly, we confirmed an increase in TFAM protein levels, following MOTS-c treatment in 293 T cells as well (Fig. S1A). An increase in protein levels of NRF1, a parallel activator of TFAM alongside PGC1α, was observed in U-2 OS cells as well (Fig. 2B). Next, we observed a strong increase in the NRF1 and TFAM downstream target, COX4, a nucleus-encoded subunit of mitochondria. There was a greater than eightfold increase in COX4 protein levels (p < 0.001) in U-2 OS cells following MOTS-c treatment (100 μM) (Fig. 2C). However, protein levels of PGC1α were not significantly higher (statistically) after MOTS-c treatment (p = 0.0564), yet some increase (> twofold) was observed in the higher treatment group (100 μM) (Fig. 2D). A similar trend in PGC1α protein levels, following MOTS-c treatment, was observed in 293 T cells as well (Fig. S1B). The protein levels of TOMM20 (Translocase of Outer Mitochondrial Membrane 20), a mitochondrial protein import receptor, remained unchanged after MOTS-c treatment in U-2 OS cells (Fig. 2E).

### MOTS-c treatment depletes mitochondrial number and density

**Figure 3:**
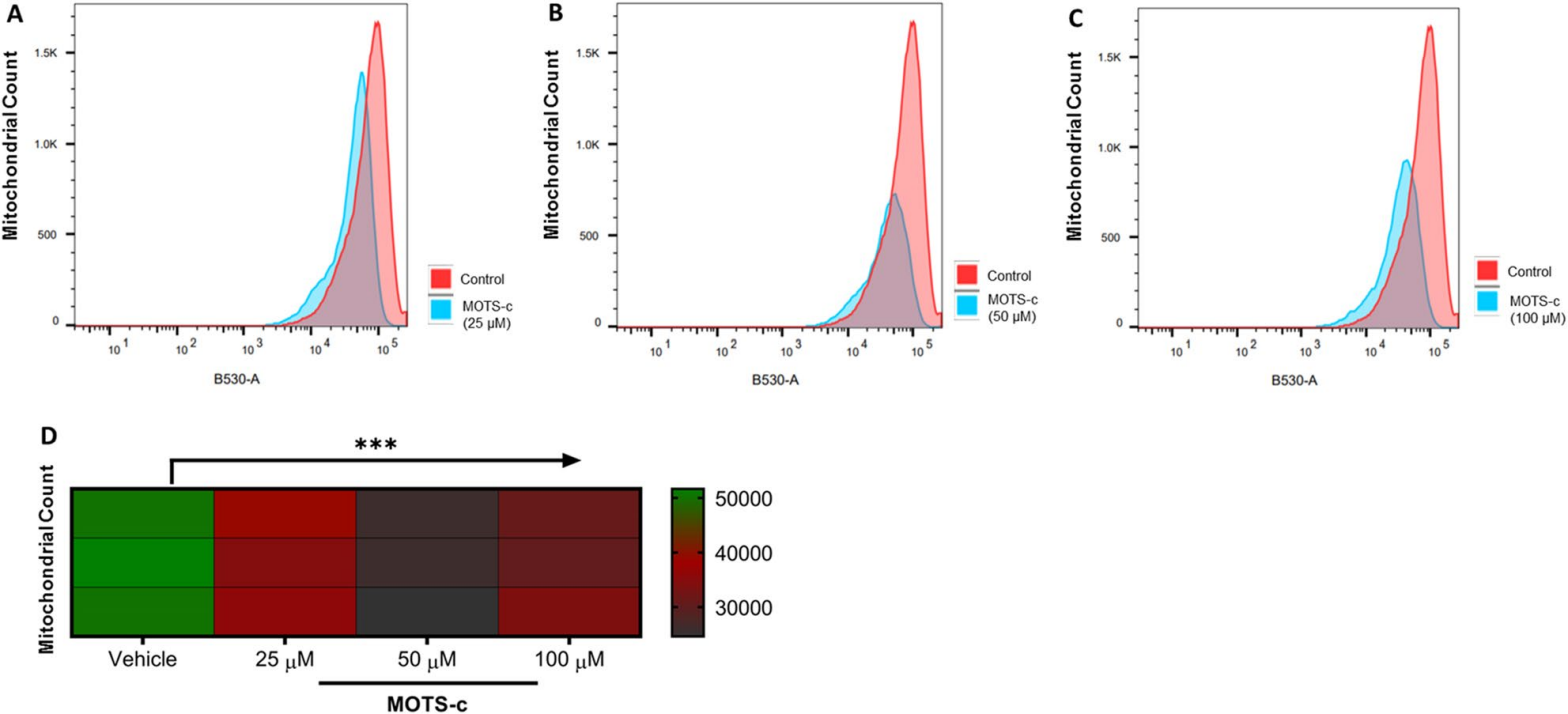
Treatment of U-2 OS cells with MOTS-c depleted the total number of mitochondria as measured by MitoTracker Green. Representative flow cytometry plots (**A**–**C**) show the intensity of MitoTracker Green in vehicle or MOTS-c and (**D**) Heatmap indicates changes in mitochondrial number *w.r.t.* vehicle control. U-2 OS cells grown in Dulbecco’s modified Eagle’s medium (DMEM) supplemented with 10% fetal bovine serum (FBS) plus 1% pen-strep were treated with vehicle (Nuclease-Free Water) or MOTS-c (25, 50 or 100 μM) for 48 h and flowcytometry was performed to quantify the number of mitochondria using MitoTracker Green, which localizes to mitochondria regardless of mitochondrial membrane potential. Data are means ± SEM of three experiments. Statistical analysis was conducted using ordinary one-way ANOVA followed by Dunnett's multiple comparisons test vs vehicle; results are presented as ***p < 0.001 vs. vehicle.

**Figure 4:**
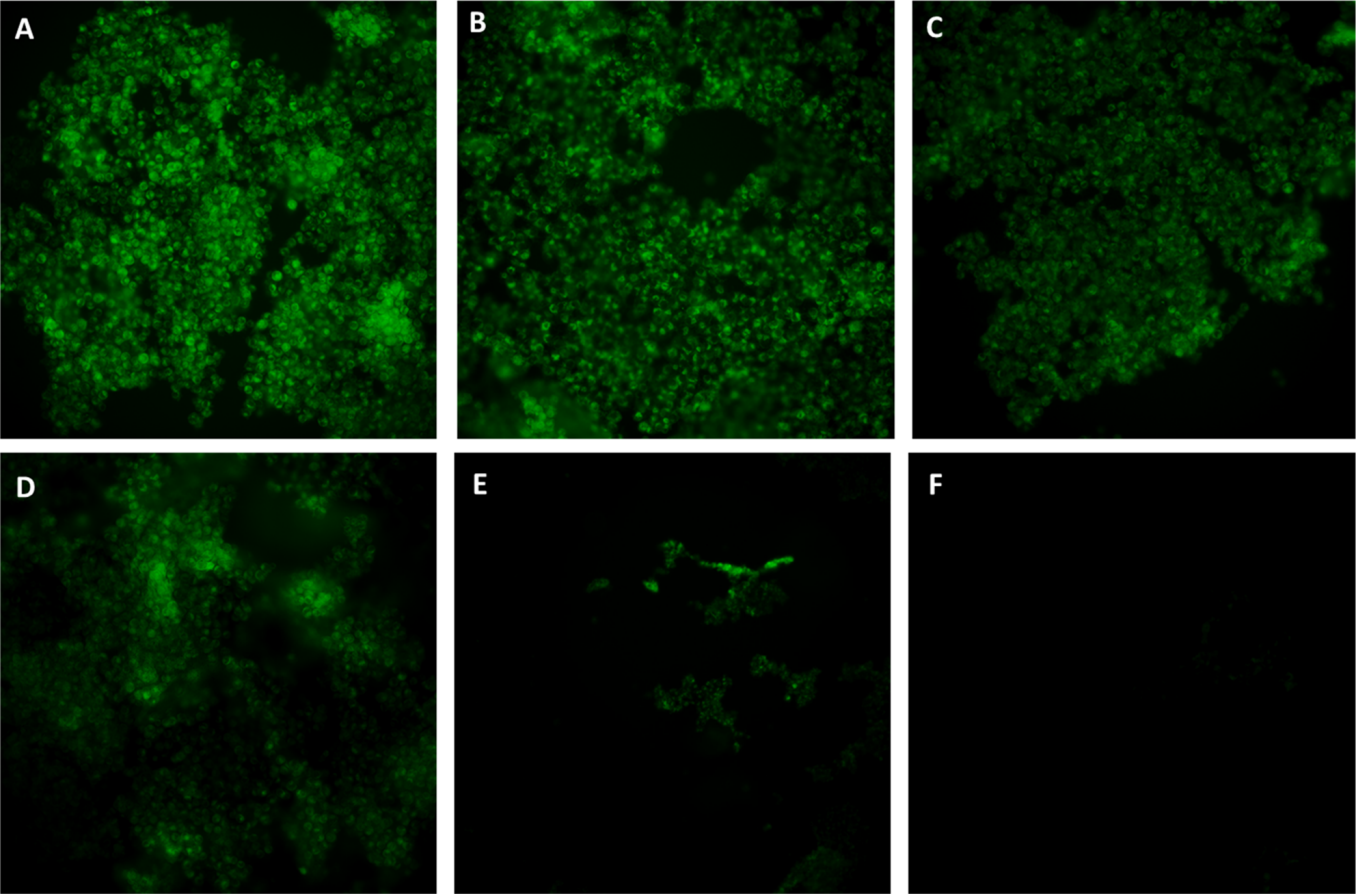
Treatment of U-2 OS cells with MOTS-c decreased the total number of mitochondria as measured by fluorescent microscopy using MitoTracker Green. Representative fluorescent microscopic images of U-2 OS cells treated with (**A**) vehicle (Nuclease-Free Water) and (**B**–**D**) MOTS-c after staining with MitoTracker Green FM (200 nM) (**E**) negative control: chloramphenicol (1 μM) and (**F**) assay control or probe control (no MitoTracker Green FM). There was depletion in MitoTracker Green intensity following increase in MOTS-c concentration (**B**) 25 μM (**C**) 50 μM and (**D**) 100 μM. U-2 OS cells grown in Dulbecco’s modified Eagle’s medium (DMEM) supplemented with 10% fetal bovine serum (FBS) plus 1% pen-strep were treated with vehicle (Nuclease-Free Water) or MOTS-c (25, 50 or 100 μM) for 48 h and fluorescent microscopy was performed to quantify the number of mitochondria using MitoTracker Green, which localizes to mitochondria regardless of mitochondrial membrane potential.

Following the confirmation of increased protein and mRNA expression of biomarkers of mitochondrial biogenesis, we measured the number of mitochondria using MitoTracker™ Green FM probe. With its ability to exhibit fluorescence only in the lipid environment of mitochondria, the number of mitochondria can be measured using flow cytometry. Contrary to our expectations, we observed a sharp decline in the number of mitochondria after MOTS-c treatment (p < 0.001) in U-2 OS cells (Fig. 3A–D). The decrease in the number of mitochondria was concentration-dependent, as an increase in the MOTS-c concentration led to a stark decline in mitochondrial number (Fig. 3A–C). Among the different MOTS-c treatments, the 50 μM group exhibited the strongest reduction in mitochondrial number(s) compared to the control group (Fig. 3D). An average of ~ 40% decrease in the mitochondrial count was observed across all the MOTS-c treatment groups. We then confirmed the decline in mitochondrial number(s) by fluorescent microscopy as well. The microscopy analysis using MitoTracker™ Green FM dye showed the depleted density of mitochondria in U-2 OS cells (Fig. 4A–D). Similar, to flow cytometry, fluorescence microscopy also showed a concentration-dependent decline in inflorescence intensity, indicative of mitochondrial number in U-2 OS cells. The microscopy results were validated by relevant negative and assay controls as well (Fig. 4E,F). However, the staining of cells with COX4 showed no decline in mitochondrial number, which can be explained by some stress in MOTS-c treated cells and different modes of action as MitoTracker™ Green, unlike COX4, exhibits fluorescence only within the lipid environment of mitochondria (Fig. S4).

### MOTS-c treatment initiates mitochondrial fusion

**Figure 5:**
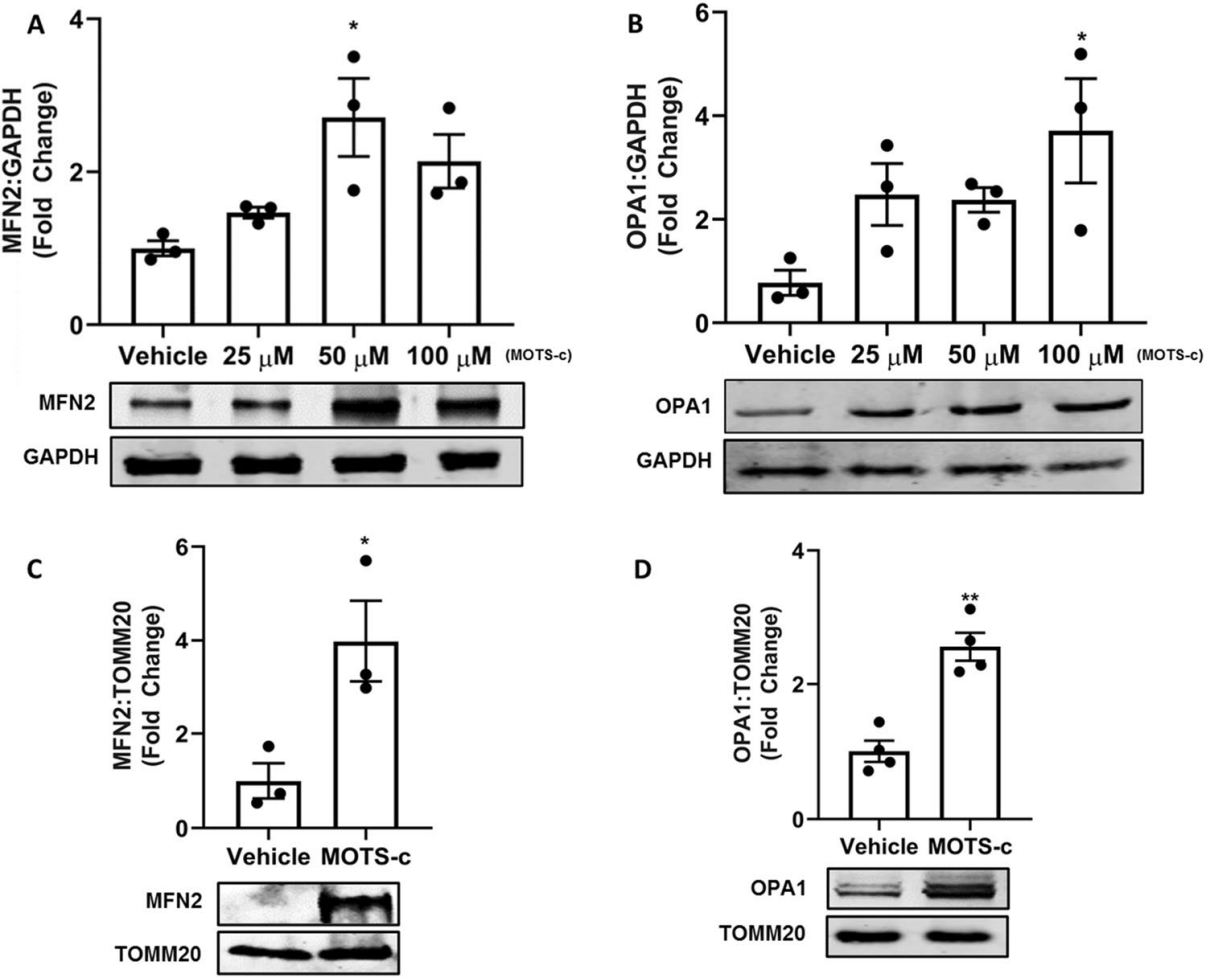
Treatment of U-2 OS cells with MOTS-c initiated mitochondrial fusion. Treatment with MOTS-c increased MfN2 and OPA1 protein expression in (**A**,**B**) whole cells and (**C**,**D**) mitochondria. U-2 OS cells grown in Dulbecco’s modified Eagle’s medium (DMEM) supplemented with 10% fetal bovine serum (FBS) plus 1% pen-strep were treated with vehicle (Nuclease-Free Water) or MOTS-c (25, 50 or 100 μM) for 48 h and western blot was performed to quantify the protein expression level of selected biomarkers. Mitochondria were extracted from cells treated with 100 μM MOTS-c only. GAPDH was used as the internal control for whole cell analysis while TOMM20 was used as the mitochondrial control. All full-length blots are presented in Supplementary Figure WB1. Data are means ± SEM of three to four experiments. Statistical analysis was conducted using ordinary one-way ANOVA followed by Dunnett's multiple comparisons test vs vehicle; results are presented as *p < 0.05, and **p < 0.01 vs. vehicle.

**Figure 6:**
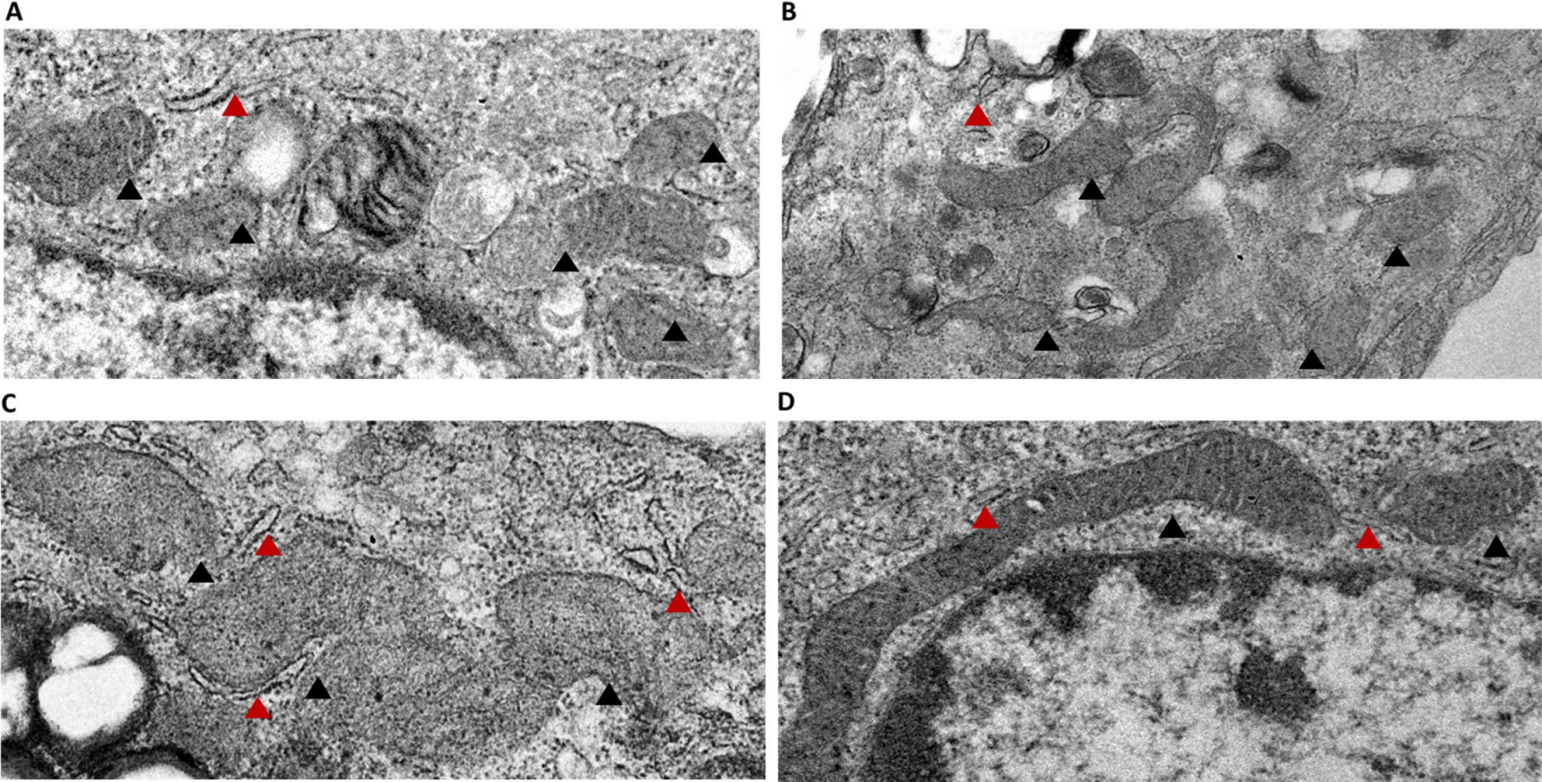
Transmission electron microscopy showing the effect of (**A**) Vehicle and MOTS-c treatment (**B**) 25 μM MOTS-c (**C**) 50 μM MOTS-c and (**D**) 100 μM MOTS-c on mitochondrial morphology. The U-2 OS cells were grown in Dulbecco’s modified Eagle’s medium (DMEM) supplemented with 10% fetal bovine serum (FBS) plus 1% pen-strep were treated with vehicle (Nuclease-Free Water) or MOTS-c (25, 50 or 100 μM) for 48 h and electron microscopy analysis was performed as described in methodology. The black triangles point towards the mitochondria while the red triangles point towards endoplasmic reticulum.

Next, to explain the depleted number of mitochondria despite the activation of PGC1α mRNA and its increased protein levels, we investigated the culmination of mitobiogenesis to mitochondrial fusion. We found that MOTS-c treatment activates mitofusin-2 (MFN2) and optic atrophy 1 (OPA1), the critical mediators of outer and inner mitochondrial membrane fusion^28^. Treatment of U-2 OS cells with MOTS-c (50 and 100 μM) significantly increased the levels of MFN2 in U-2 OS cells (p < 0.05) (Fig. 5A). Similarly, an increase in OPA1 was observed following MOTS-c treatment (100 μM) in U-2 OS cells (p < 0.05). These findings were confirmed with similar results in 293 T cells, indicating an increase in MFN2 and OPA1 following MOTS-c treatment (50 and 100 μM) (Fig. S1C,D). Further, we extracted mitochondria to confirm the increase of the two GTPases tethered to the mitochondrial membrane. Our results showed a significant increase in both MFN2 and OPA1 in the extracted mitochondria as well (p < 0.01; p < 0.05) (Fig. 5C,D). Further, the electron microscopy analysis confirmed the activation of mitochondrial fusion by MOTS-c treatment in U-2 OS cells. Compared to the vehicle (Fig. 6A), there was depletion in the number of mitochondria following MOTS-c treatment (Fig. 6B–D). The exact quantified number of mitochondria after each treatment are presented in Fig. S3. Also, the initiation of mitochondrial fusion can be seen beginning at 48 h at different treatment concentrations of MOTS-c (Fig. 6B–D). At the 25 μM MOTS-c treatment, long mitochondria begin to emerge after 48 h, although the increase of MFN2 and OPA1 levels were not significant in U-2 OS cells (Fig. 5A–D). At the 50 and 100 μM MOTS-c treatment, the presence of longer and fusing mitochondria were observed which supported a significantly increased MFN2 and OPA1 levels in U-2 OS cells (Fig. 5A–D). Interestingly, prospective tethering of mitochondria and endoplasmic reticulum (ER) is observed at the 50 and 100 μM MOTS-c treatment (Fig. 6C,D). These results confirm the activation of mitochondrial fusion following MOTS-c treatment, a novel function of MOTS-c and mitochondrial retrograde signaling.

### MOTS-c mediated glucose uptake is dependent on mitochondrial fusion

**Figure 7:**
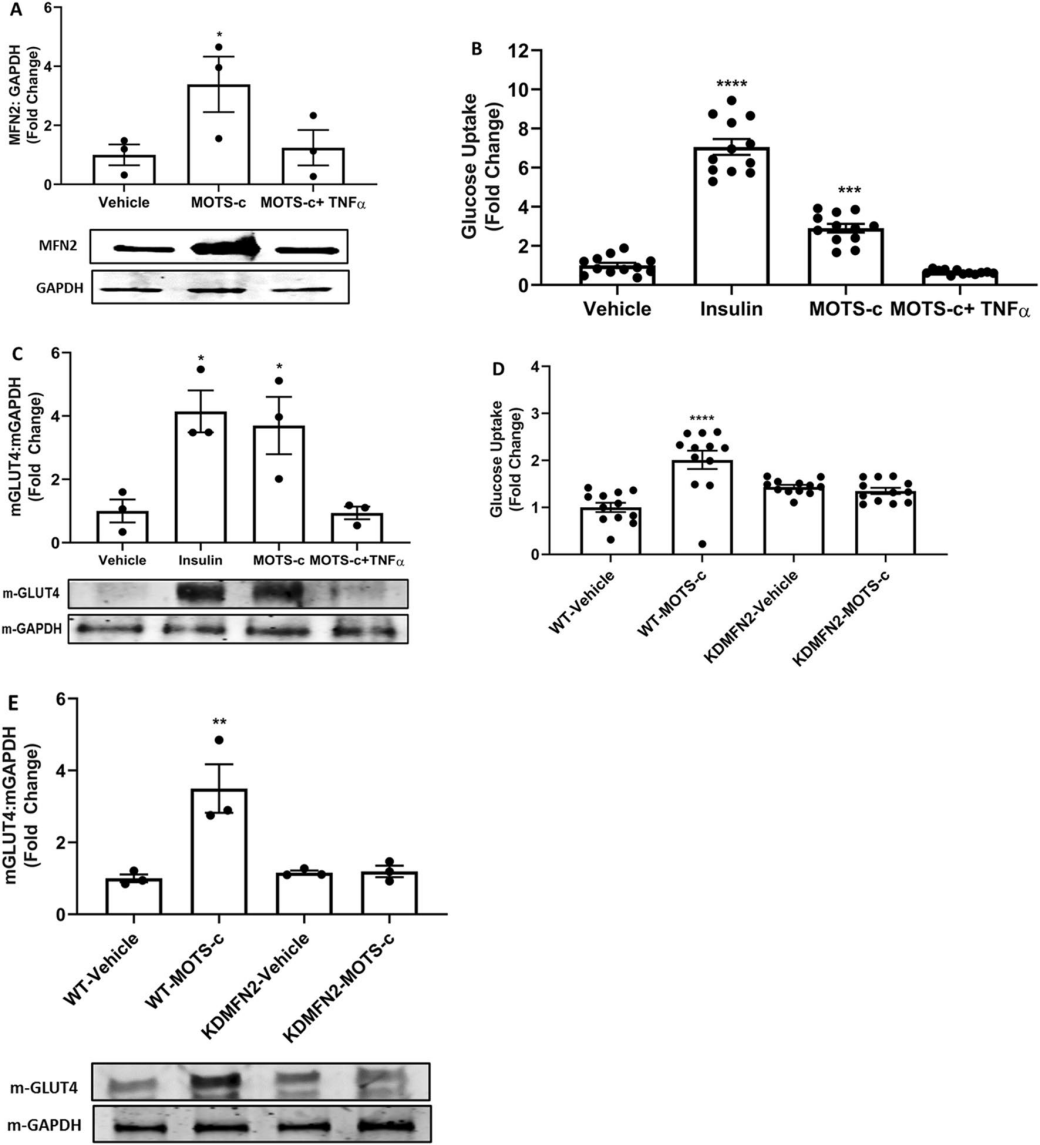
Mitofusion underlines the ability of mitochondrial peptide MOTS‐c to prompt GLUT4 translocation. MOTS-c (100 μM) treatment prompted (**A**) MFN2 increase in D12 adipocytes (**B**) MFN2 dependent glucose uptake and (**C**) MFN2 dependent membrane GLUT4 translocation in D12 adipocytes was abrogated by TNFα (10 ng/mL), a known inhibitor of mitofusion, and in (**D**,**E**) siRNA KD of MFN2. D12 pre-adipocytes were differentiated into brown adipocytes in DMEM/F12 media supplemented with 15% fetal bovine serum (FBS), 1% l-Alanyl-l-Glutamine plus 1% pen-strep to detect glucose uptake (Glucose Uptake-Glo™ Assay) and western blot was performed to quantify the protein expression level of GLUT4. GAPDH was used as the internal control for western blot. Data are means ± SEM of three to twelve experiments. All full-length blots are presented in Supplementary Figure WB1. Statistical analysis was conducted using ordinary one-way ANOVA followed by Dunnett's multiple comparisons test vs vehicle; results are presented as *p < 0.05, **p < 0.01 and ****p < 0.0001 vs. vehicle.

First, we studied the effect of MOTS-c treatment on MFN2 protein levels in D12 adipocytes. Then we checked if TNFα, an inhibitor of mitochondrial fusion, can nullify the MFN2 increase by MOTS-c in adipocytes. TNFα is known to decrease the levels of MFN2 in smooth muscle cells and fibroblasts^28,29^. However, MFN2^−/−^ macrophages, the expression of TNFα and IL-1β was reduced, indicating cell type as a key factor^30^. Our results showed that MOTS-c significantly increased the protein levels of MFN2 in D12 adipocytes, which was abrogated by TNFα (10 ng/mL) co-treatment (Fig. 7A). MFN2 increase showed a vital finding that MOTS-c can trigger mitochondrial fusion in adipocytes, the cells vitally connected to the metabolic homeostasis^31^. Next, we conducted a glucose uptake assay in D12 adipocytes and compared the glucose uptake potential of MOTS-c in the presence and absence of TNFα. We used adipocytes as with the development of insulin resistance, GLUT4 expression is first downregulated selectively in adipose tissue but not in skeletal muscle and later extends to muscles^32,33^. Our results showed a significantly robust increase in glucose uptake by MOTS-c (100 μM), mimicking insulin activity in D12 cells (p < 0.001). However, the TNFα co-treatment completely abrogated the glucose uptake ability of MOTS-c in D12 adipocytes in vitro (Fig. 7B). We also investigated if analogous to glucose uptake activity, GLUT4 translocation by MOTS-c was mitochondrial fusion dependent. Our results showed that MOTS-c, in line with glucose uptake, translocated GLUT4 vesicle from the cytosol to the membrane of D12 adipocytes (p < 0.05) (Fig. 7C). However, in the presence of TNFα, the ability of MOTS-c to translocate GLUT4 to the cell membrane was entirely abolished (Fig. 7B). These results confirmed the mitochondrial fusion dependent metabolic ability of MOTS-c in cultured adipocytes.

## Discussion

Discovered around 60 years ago^34^, the mitochondrial genome is a bacteria-like round DNA containing previously unknown short open reading frames, encoding for polypeptides with biological activity. Humanin was the first conserved mitochondrial polypeptide discovered in 2001 with metabolic and cytoprotective activities^35^. The discovery of humanin signified a paradigm shift in mitochondrial studies, which lead to the discovery of many small humanin like peptides 1–6 (SHLPs)^36^ and MOTS-c^3^. MOTS-c is proposed to exhibit the “Crabtree effect” as it stimulates cellular glucose uptake while suppressing respiration (similar to AICAR and metformin)^37^. Unfortunately, little is known about the impact of MOTS-c on the mitochondrial mechanism(s) and their consequential relation to its metabolic activity. We explored whether MOTS-c influence mitochondrial dynamics to boost metabolism via increase in number mitochondria or by generation of specialized mitochondria.

Our findings highlight an active role for MOTS-c in supporting mitochondrial biogenesis, mitochondrial fusion, and eventually metabolism. Mitochondria are vital players in metabolic homeostasis and orchestrate initiation or downregulation of mitochondrial biogenesis, to achieve their desired metabolic goals. The impairment of mitochondrial biogenesis is correlated to metabolic diseases such as type 2 diabetes and obesity, the pharmacological strongholds of MOTS-c^3,7,38^. Our experiments showed substantial activation of mitochondrial biogenesis factors by MOTS-c at basal conditions in cells (Figs. 1, 2). The most striking change following MOTS-c treatment was parallel enrichment of nuclear (PGCα and NRF1) and mitochondrial genes (ND1, ND6, and ATP8) involved in mitochondria biogenesis (Fig. 1B–D). However, our immunoblot data failed to show upregulation of PGC1α, but displayed a strong expression of confirmatory markers of mitochondrial biogenesis, NRF1, TFAM, and COX4 (Figs. 2A–D; S1A,B). These findings indicate that for MOTS-c, PGC1α is probably dispensable for mitochondrial biogenesis, an observation similar to PGC1α independent exercise-induced mitochondrial biogenesis in skeletal muscle^39^. Interestingly, MOTS-c also exerts exercise mimetic effects in rodents^9^, thus explaining the lack of PGC1α expression at the protein levels^9,39^. Likewise, PGC-1α is also dispensable for mitochondrial biogenesis induced by rosiglitazone, a diabetes drug^40^. Our initial findings showed that MOTS-c treatment induced mitochondrial activation and initiation of mitochondrial biogenesis. Next, we conducted flow cytometry and fluorescence microscopy experiments using MitoTracker™ Green FM probe to measure the number of mitochondria. These experiments led to surprising results and insights into MOTS-c activity. When analyzing mitochondrial number, we expected increased mitochondrial number linked to increased transcription factors, such as PGC1α, NRF1, TFAM, and COX4. Remarkably, despite the activation of these pathways, we found a sharp decrease in the total number and density of mitochondria, following treatment with MOTS-c (Figs. 3, 4). These results suggested that the initiation of mitochondrial biogenesis by MOTS-c culminates in another event, the activation of a synchronized system of mitochondrial biogenesis and mitochondrial fusion, resulting in fewer mitochondrion.

**Figure 8:**
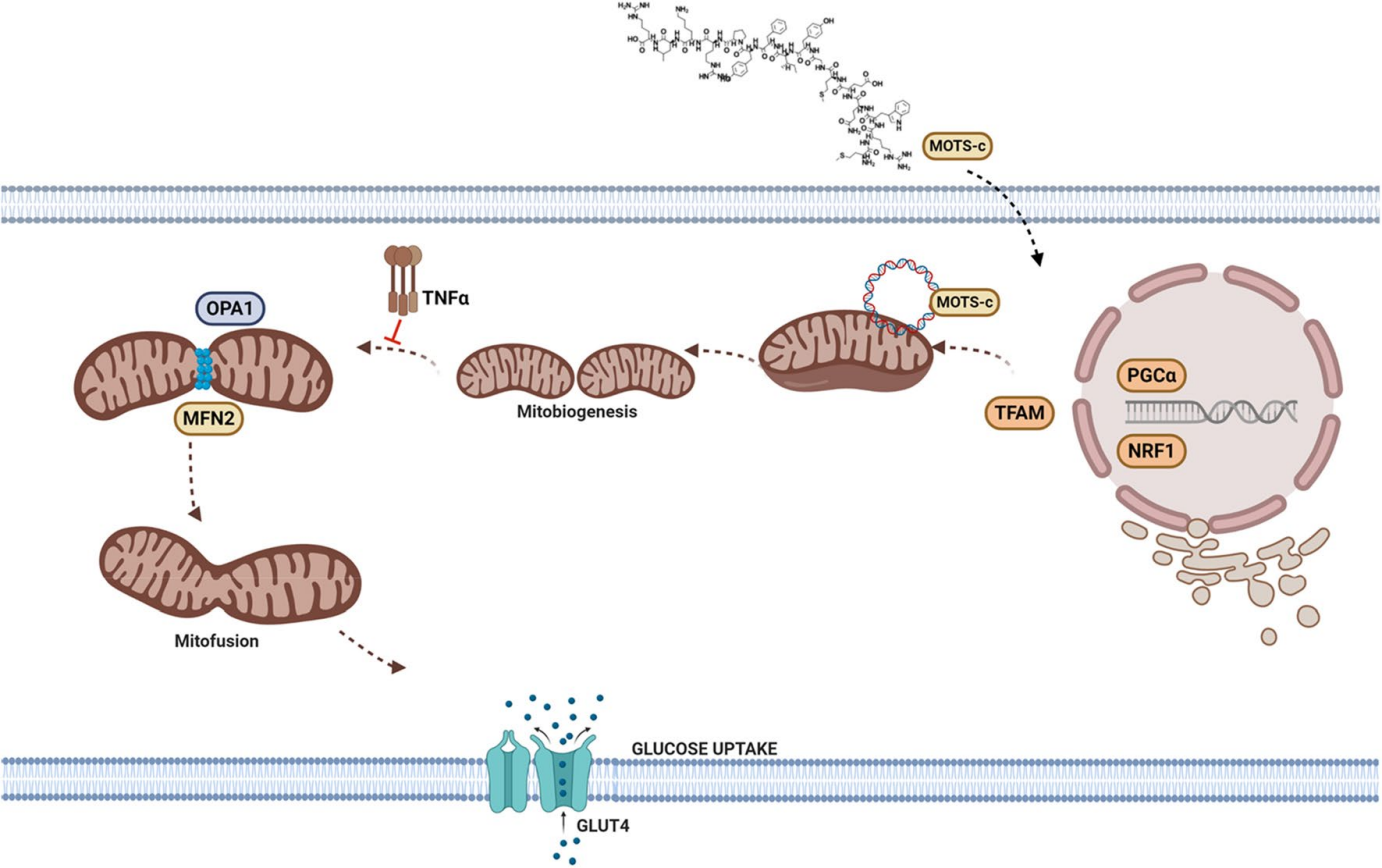
A schematic representation of mitochondrial changes and pathways underlying the ability of mitochondrial peptide MOTS‐c to prompt GLUT4 translocation.

Mitochondrial morphology, a vital determinant of cellular function, depends on the essential axis of mitochondrial fusion and fission. It is understood for more than 100 years that mitochondria fuse and divide^41^, and presently we understand the complex machinery involved in the process^42^. Mitochondrial fusion is a complex process regulated by MFN2 and OPA1, both controlled by transcription factor transcriptional coactivator PGC1β causing mitochondrial elongation^42^. Our data showed a strong increase in mitochondrial fusion markers, MFN2, and OPA1 in both whole-cell extract and in the separated mitochondria of cells treated with MOTS-c (Figs. 5A–D, S1C,D). This is a dynamic observation as the fusion of mitochondria is required to maintain cellular respiration^43^. Kim and co-workers postulated in their study that the effect of MOTS-c on cellular respiration may involve non-nuclear targets, such as mitochondria^5^. Therefore, the accumulation of MFN2 and OPA1 in mitochondria resulting in mitochondrial fusion explains the effect of MOTS-c on cellular respiration. Further, loss of outer membrane mitochondria fusion, a process controlled by MFN2, results in susceptibility to insulin resistance^44^. A study previously showed an increase in mRNA levels MFN2 in the small intestine of mice fed MOTS-c (10 mg/kg) for 2 weeks^45^. This study showed anti-aging effects of MOTS-c on the skin in vivo, however, the exploration of the relation of MOTS-c and mitochondrial fusion mechanisms was not carried out. Likewise, activation of OPA1 by MOTS-c can explain its anti-inflammatory impact^13^, owing to the direct role of OPA1 in NF-κB homeostasis^46^. Also, mitochondrial fusion is positively related to increased ATP production^47^, thus explaining the increase in ATP production following after 72 h of MOTS-c treatment in HEK293 cells^3^. It is also conceivable that MOTS-c might induce a state of mild stress and increased OXPHOS triggering mitochondrial fusion. Interestingly, fasting or caloric restriction (CR) induces mitochondrial fusion, therefore it is possible that MOTS-c acts like a CR mimetic, besides, as an exercise mimetic, as known previously^9^. Further, activation of MFN2 tethers mitochondria to the ER, a juxtaposition required for efficient mitochondrial Ca^2+^ uptake^48^. Our ESM results show closely placed ER to elongated mitochondria after MOTS-c treatment in U-2 OS cells (Fig. 6C,D). This unique functionality of MFN2 and its activation by MOTS-c explains our next finding, i.e. the mitochondrial fusion dependent glucose uptake and GLUT4 translocation by MOTS-c. Ca^2+^ signaling controls insulin-stimulated glucose uptake in skeletal muscle^49^, and explain the process by which exercise ameliorates glucose homeostasis in individuals with type 2 diabetes^49,50^. Similarly, MFN2 is vital in maintaining adipocyte and related fat metabolism as its knockdown in adipose tissue of adult mice results in an obese phenotype^31^. Further, activation of MFN2, a mitochondrial fusion regulator and a cellular signal for Ca^2+^ uptake, explains the glucose uptake by MOTS-c in D12 adipocytes (Fig. 7A,B). Similar, but weaker than insulin, glucose uptake by MOTS-c was abrogated in the presence of MFN2 inhibitor TNFα (10 ng/mL) (Fig. 7B). These results reveal the mitochondrial mechanisms underlying glucose uptake by MOTS-c. Likewise, the ability of MOTS-c to trigger GLUT4 translocation was abrogated upon MFN2 inhibition by TNFα (Fig. 7C). Also, change in amino acid sequence of MOTS-c diminishes its ability to increase MFN2 expression in U-2 OS (Fig. S2). Overall, these findings reveal that MOTS-c meticulously triggers well-orchestrated mitochondrial biogenesis, culminating in mitochondrial fusion via activation of MFN2 and OPA1, a dynamic process which underlies its metabolic activity (Fig. 8).

The research findings related to metabolism are not always similar when studying in vitro and in vivo models. It will be crucial for future studies on MOTS-c to validate these findings in vivo. The in vivo physiological complexity, host-immune system, and metabolic differences between cells and animals may dictate the application of our findings. The extent to which an in vivo niche modifies mitochondrial dynamics, its relation to metabolism, and specific tissue function cannot be hypothesized in this paper. For example, metabolic by-products of MOTS-c might engage different cellular pathways in vivo. Further, choice of in vivo model systems, such as basal or pathological model systems, metabolite accumulation, and secondary or off-target effects, and design of experiments may dictate the impact MOTS-c on mitochondrial fusion in vivo. Studies on mitochondrial peptides are an exciting and growing field and will continue to reveal the diverse roles of these peptides and versatile signaling in cellular homeostasis and disease.

## Conclusion

The evolving field of mitochondria derived peptides like MOTS-c and humanin symbolizes the foreground of novel metabolism and mitochondrial findings. To the best of our knowledge, this is the first study showing the ability of MOTS-c to trigger mitochondrial fusion and explain its underlined mechanisms. In sum, our study reveals a synchronized programing of mitochondrial biogenesis and mitochondrial fusion by exogenous MOTS-c in vitro, a novel process contributing to its metabolic activity. Also, our study can enable other researchers to study novel biomarkers for examining pharmacological activities of mitochondrial peptides and similar molecules. Deciphering the details of these findings and identifying targets, and discovering new mitochondrial peptides holds therapeutic potential in the field of mitochondria, metabolism, and endocrinology.

## Methods

### Cell culture

U-2 OS (ATCC^®^ HTB-96™) and 293T (ATCC^®^ CRL-3216™) cells purchased from ATCC (American Type Culture Collection) were cultured in Dulbecco's modified Eagle's medium (DMEM; HyClone™) supplemented with 10% fetal bovine serum (FBS; Gibco™ LS26140079), l-Glutamine (Gibco™ LS25030081), MEM Non-Essential Amino Acids Solution (Gibco™ LS11140050), and 100 units/mL penicillin–streptomycin (Gibco™ 15140122) at 37 °C and 5% CO_2_ in an incubator. D12 (ATCC^®^ CRL-3280™) mouse pre-adipocytes (Beige adipocytes) were differentiated into brown adipocytes in DMEM/F12 media with supplementation similar to U-2 OS and 293 T cells plus 1% l-Alanyl-l-Glutamine. All cells were initially cultured in Corning^®^ T-75 flasks (catalog #430641) in a subcultivation ratio of 1:6. The cell medium was replaced every 48–72 h and all experiments were conducted using cells between passages 4–8.

### Peptide synthesis

The synthesized MOTS-c (H-MRWQEMGYIFYPRKLR-OH) with a minimum purity of 99% was obtained from GenScript (Piscataway, NJ, USA). Synthesized MOTS-c was dissolved in PBS (Gibco™ 10010023) at a stock concentration of 100 mM, aliquoted, and stored at − 80 °C until further usage.

### Experimental conditions

Experiments with U-2 OS cells were performed in Standard Tissue Culture Dishes (Falcon™ 08-772E). Cells were treated either by MOTS-c (25, 50, or 100 µM) or vehicle (PBS) for 48 h. Following treatment, cells were lysed in 300 μL RIPA buffer supplemented with cOmplete™ protease inhibitor (Roche Life Science, 11697498001) and PhosSTOP™ protease inhibitor cocktails (Roche Life Science, 4906845001). Once extracted, the cell lysates were stored at − 20 °C until further usage.

### Immunoblotting

Immunoblotting was performed according to our previous study^51^. Primary antibodies used in immunoblotting including TFAM (ab138351), COX4 (ab202554), NRF1 (ab175932), PGC1α (ab54481), TOMM20 (ab186735), MFN2 (ab124773), GLUT4 (ab654) and GAPDH (ab8245) were purchased from Abcam (Abcam Inc, Toronto, Canada). The OPA1 (NBP2-59770SS) antibody was obtained from Novus Biologicals (Toronto, Canada). All the antibodies were used at a dilution of 1:1000.

### RNA extraction and RT-PCR

Total RNA was isolated from cells by extraction with TRIzol reagent, and the purity of the RNA preparations was assessed using the A260/280 ratio. We performed RT-PCR using Fast SYBR Green PCR Master Mix (Applied Biosystem, Burlington, ON, Canada) and the amplification conditions were as follows: initial denaturation at 95 °C for 10 min followed by 35 cycles of 10 s at 95 °C, 15 s at 60 °C, and 10 s at 72 °C. as described in detail previously^52^. The primers used were PGC1α (FP: AGCCTCTTTGCCCAGATCTT RP: GGCAATCCGTCTTCATCCAC), NRF1 (FP: CGGTATGCAACAGGACATTG RP: ACTGGTTGGGGTCTTCTGTG), ND1 (FP: ATCAGGGTGAGCATCAAA RP: TTCGGTTGGTCTCTGCTA), ND6 (FP: ACAGCACCAATCCTACCT RP: ATTGTTAGCGGTGTGGTC), ATP8 (FP: CCACTGTAAAGCTAACTTAGC RP: GTTAGG GGTCATGGGCTG) and GAPDH (FP: GAAGGTGAAGGTCGGAGTC RP: GAAGATGGTGATGGGATTTC).

### Flowcytometry

The mitochondrial content was measured using the MitoTracker™ Green FM (Invitrogen™, M7514). The selection of MitoTracker™ Green FM is based on its ability to be essentially nonfluorescent in aqueous solutions and exhibit fluorescence once it accumulates in the lipid environment of mitochondria. Therefore, the false-positive results are negligible, enabling a correct count of mitochondria via bright green, fluorescein-like fluorescence. Briefly, cells were cultured and treated as indicated, trypsinized, spun down at 400 rpm, and resuspended in buffer made from PBS and FBS (3:1) with MitoTracker™ Green FM at a concentration between 100–400 nM. After incubating between cells with dye for 15–30 min at 37 °C, mitochondria were analyzed using the BD FACSCanto™ (BD FACS Canto II) flow cytometry cell analyzer (BD Biosciences, San Jose, CAL, USA). The FlowJo software was used for the analysis of flow cytometry data (Tree Star, Inc. OR, USA).

### Fluorescence imaging

All fluorescence imaging experiments were performed on a Zeiss Colibri Fluorescence Microscope (Carl Zeiss Canada Ltd., Toronto, Canada). Cells were grown at a density of 100 cells per well on chamber slides Nunc^®^ Lab-Tek^®^ Chamber Slide™ system (Millipore-Sigma, C7182). Cells were treated as described earlier and mitochondria were imaged using MitoTracker™ Green FM (Invitrogen™, M7514). The filters for excitation: 490 nm and emission: 516 nm were used according to the dye.

### Transmission electron microscope (TEM)

Cells were treated with Vehicle or MOTS-c for 48 h and then fixed using TEM fixing solution: 2% PFA + 2.5% GTA in 0.1 M phosphate buffer. All experiments for TEM were conducted in 100 mm cell culture dishes and a minimum of 4 × 10^6^ cells were collected for each sample (n = 4). After trypsinizing and fixing the cells, cells were kept at 4 °C till further analysis. For the development of EM images, the cell pellet(es) was rinsed with 0.1 M phosphate buffer (10 min × 3), postfix was conducted with 1% OsO_4_/0.1 M phosphate buffer for 1 h. The pellets were rinsed with H_2_O (10 min × 3) and stained with 1% uranyl acetate/H_2_O overnight. Samples were rinsed with H_2_O, (10 min × 3) and stained with 1% lead nitrate/aspartate solution 30 min at 60 °C water bath. The samples were then washed with H_2_O (10 min × 3). Next, samples were dehydrated with 30%, 50%, 70% and 95% ethanol (10 min × 1) and 100% ethanol (10 min × 3). Next, a graded infiltration procedure was performed using: 25% Spurr’s (2 h); 50% Spurr’s (2 h); 75% Spurr’s (4 h) and 100% Spurr’s (24 h with 3–4 changes). Following graded infiltration, the specimen was transferred into a flat mold with fresh Spurr’s resin, polymerized at 70 °C oven 20–24 h, and a minimum of 8 images were collected for each treatment group using Hitachi H-7650 Transmission Electron Microscope (Hitachi, Dallas, TX, USA)^53^.

### Mitochondrial fusion inhibitor treatments

TNFα is a known inhibitor of mitochondrial fusion and downregulates expression of mitofusion genes like MfN2^28^. Therefore, for inhibition of mitochondrial fusion, cells were incubated in the presence or absence of TNFα (10 ng/mL) for 24 h. Other subsequent experiments were performed as specified in the relevant sections.

### Glucose uptake assay

Glucose uptake assay in D12 adipocytes was performed Glucose Uptake-Glo™ Assay based on the detection of 2-deoxyglucose-6-phosphate (2DG6P) (Promega, WI, U.S.A). Briefly, the D12 cells were plated at 20,000 cells per 100 µl media in a 96-well plate. Adipocytes were grown to ~ 70% confluency at 37 °C in 5% CO_2_ with medium replacement every 48 h. The treatment groups were divided into 1) Vehicle: receiving PBS 2) Insulin: receiving insulin (100 nM) for 6 h 3) MOTS-c (100 μM) for 48 h and 4) MOTS-c + TNFα receiving co-treatment of MOTS-c (100 μM) and TNFα (10 ng/mL) for 48 h. The glucose uptake was measured using SpectraMax M3 spectrophotometer (Molecular devices, CA, USA) and the results were expressed as change in glucose uptake (fold change w.r.t. vehicle).

### siRNA experiments

For KD experiments, the growth medium of D12 adipocytes was replaced 1 h before transfection with antibiotic-free medium. Control siRNA (4390843) or Mfn2-siRNA (s100687, 4390771) were added to the transfection mixture to a final concentration of 50 nM (Life Technologies Inc, Burlington, ON). D12 adipocytes were transfected using Lipofectamine™ 2000 Transfection Reagent (Life Technologies Inc, Burlington, ON) according to the supplier’s instructions. KD experiments were performed 2 days following transfection.

### Membrane and mitochondria extraction

The subcellular fractionation of U-2 OS cells was performed to extract mitochondria and membrane proteins according to a previously described metods^54,55^.

### Statistical analysis

All experiments were repeated at least three (n = 3–9). All the data were analyzed using GraphPad Prism software (GraphPad Software Inc., CA, USA) and expressed as the mean ± standard error (SE). One-way ANOVA and Dunnett's multiple comparisons test were used for statistical analysis and a value of *p* < 0.05 was considered statistically significant.

## Supplementary Information

Supplementary Information.

## Data Availability

The data generated and analyzed during the current study are included in this article and detailed datasets are available on request from the corresponding author.
