# Supplementary material for: Mitofusion is required for MOTS‐c induced GLUT4 translocation
Source: Sci Rep. 2021 Jul 12;11:14291. doi: 10.1038/s41598-021-93735-2 (PMC8275580; doi:10.1038/s41598-021-93735-2)

**Supplementary Information**

**Mitofusion is required for MOTS‐c induced GLUT4 translocation**

**Khushwant S. Bhullar^1,2^, Nan Shang^1^, Evan Kerek^2^, Kaiyu Wu^1^, and Jianping Wu^1*^**

^1^Department of Agricultural, Food, and Nutritional Science, University of Alberta, Edmonton, AB, Canada; ^2^Department of Pharmacology, University of Alberta, Edmonton, AB, Canada.

**
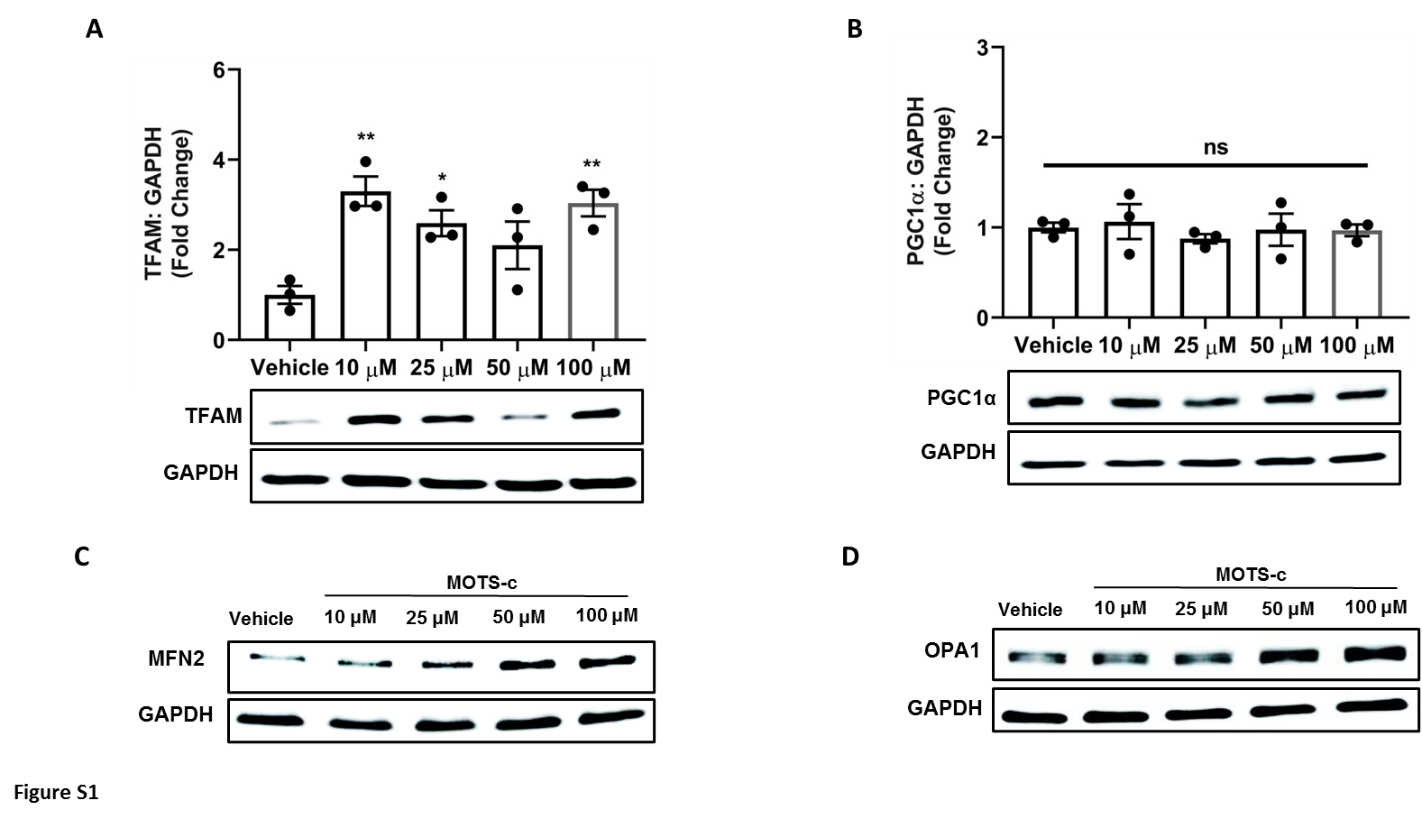
**

**Figure S1**: Treatment of 293T cells with MOTS-c upregulated biomarkers of mitochondrial biogenesis and mitofusion. MOTS-c treatment increased protein expression of (A) TFAM (B) PGC1α (C) MFN2 and (D) OPA1 protein expression. 293T cells grown in Dulbecco’s modified Eagle’s medium (DMEM supplemented with 10% fetal bovine serum (FBS) were treated with vehicle (Nuclease-Free Water) or MOTS-c (10, 25, 50 or 100 μM) for 48 h and western blot was performed to quantify the protein expression level of selected biomarkers. GAPDH was used as the internal control. Data are means ± SEM of three experiments. Statistical analysis was conducted using ordinary one-way ANOVA followed by Dunnett's multiple comparisons test vs vehicle; results are presented as *p<0.05, **p<0.01 and ****p<0.0001 vs. vehicle.

**
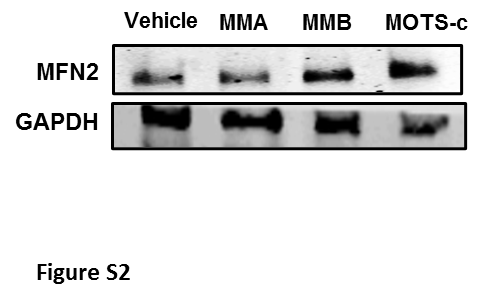
**

**Figure S2**: Comparative impact of MOTS-c and related peptides on MFN2 expression in 293T cells. 293T cells grown in Dulbecco’s modified Eagle’s medium (DMEM supplemented with 10% fetal bovine serum (FBS) were treated with vehicle (Nuclease-Free Water), modified A MOTS-c (MRWQEMYIFYPRKLR; 100 μM), modified B MOTS-c (MQEMGYFYPKL; 100 μM) or MOTS-c (100 μM) for 48 and western blot was performed to quantify the protein expression level of MFN2. GAPDH was used as the internal control.


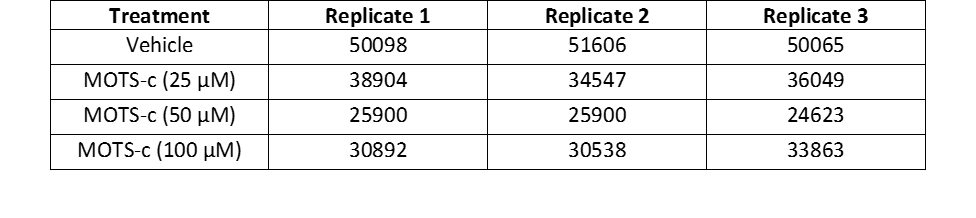


**Figure S3**: The change in number of mitochondria as measured by MitoTracker Green. The cells were treated with vehicle (Nuclease-Free Water) or MOTS-c (25, 50 or 100 μM) for 48 h and flowcytometry was performed as described in the methods. The representative results (mitochondrial number) are derived from 50000 events (each replicate) measured by flowcytometry.


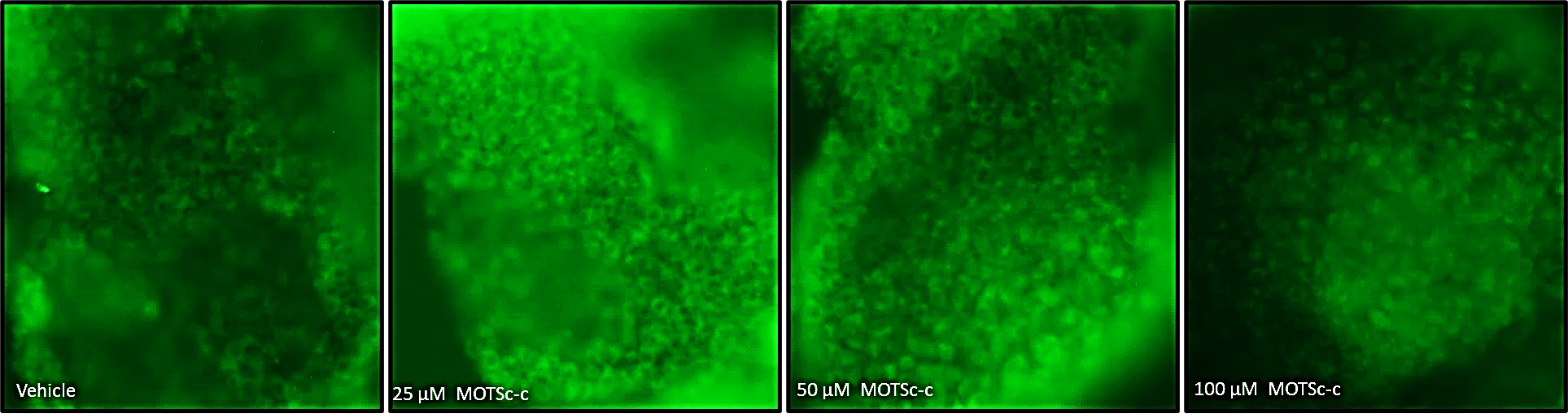


**Figure S4**: The impact of MOTS-c treatment on COX4 levels in in U-2 OS cells.


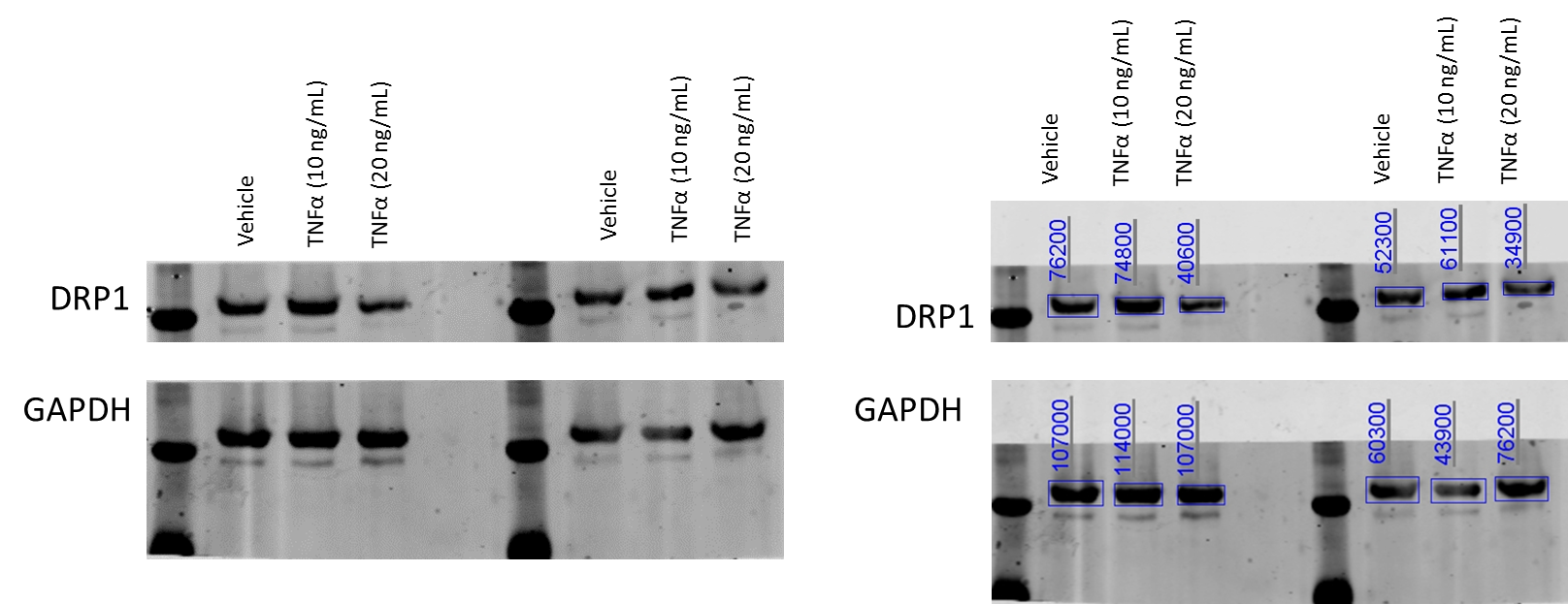


**Figure S5**: The impact of TNFα treatment on DRP1 expression in U-2 OS cells. U-2 OS cells were treated with vehicle (Nuclease-Free Water), or TNFα (10 or 20 ng/mL) and western blot was performed as described in the methods.

**Supplementary Western blots:** Immunoblots with membrane photo overlay and band density. Membranes were cut with a scalpel before probing with the detection antibody according to the protein markers to encapsulate all regions of interest as determined by the antibody reference sheet provided by the supplier. Blue dashed boxes indicate the area used for the WB presented. Control bands were included for all membranes.

**
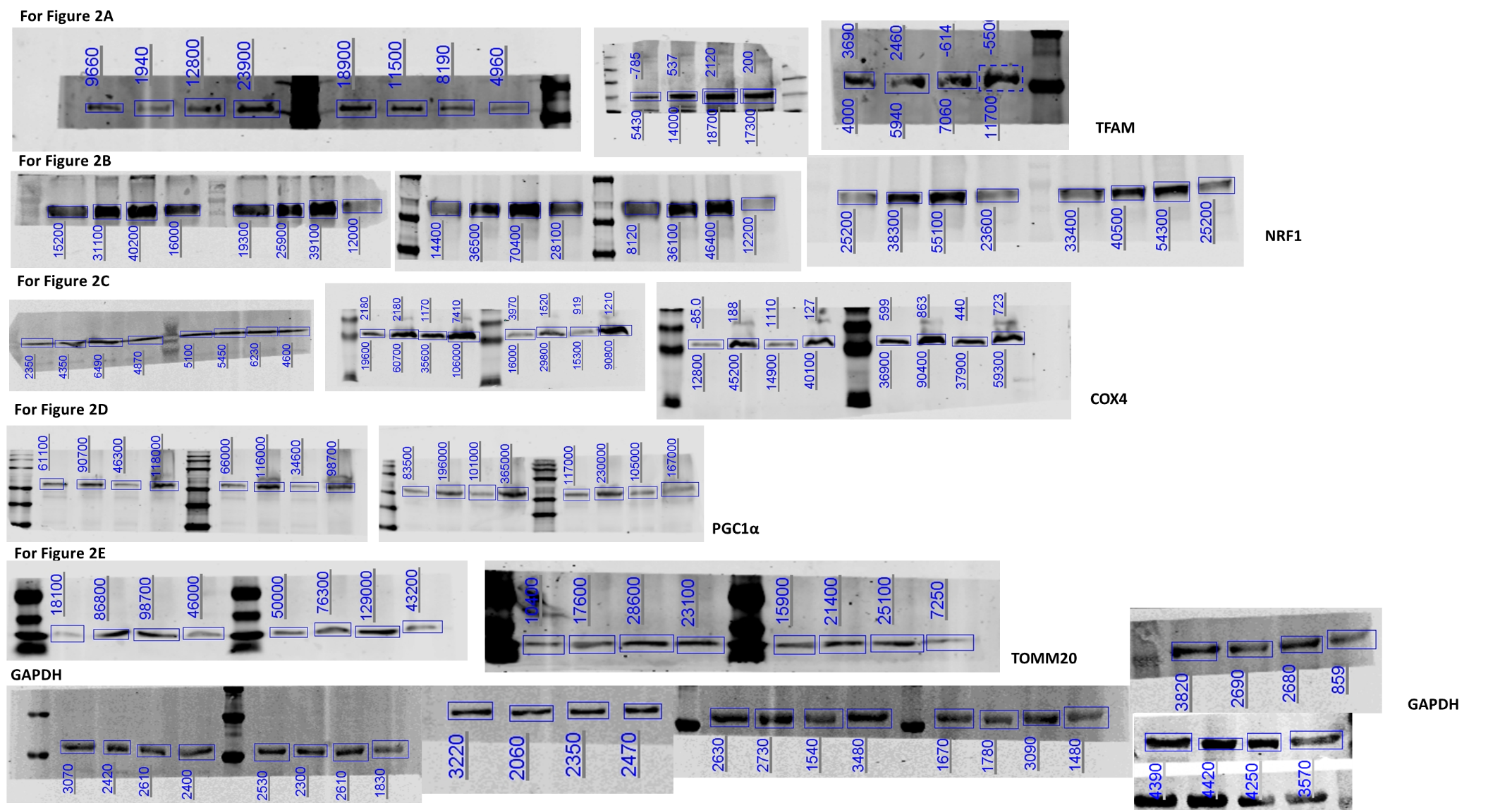
**

**
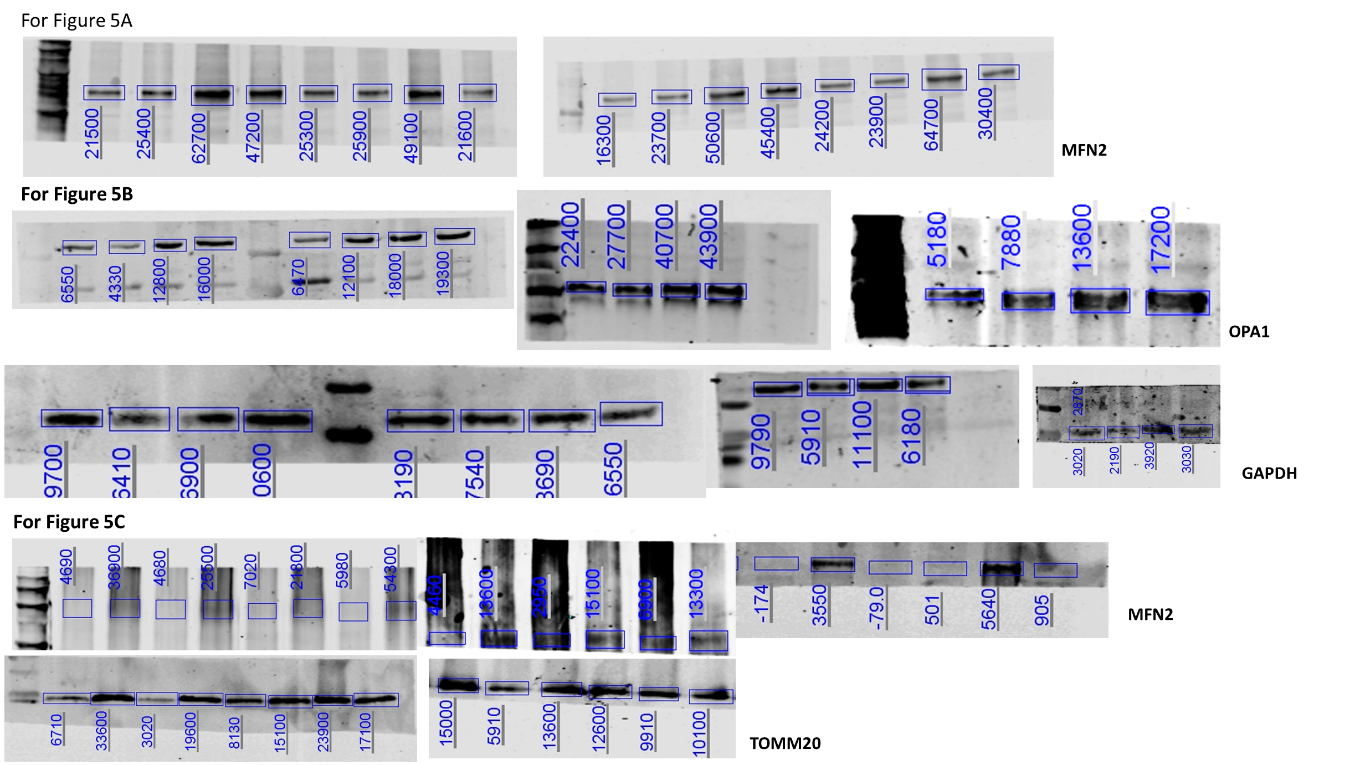
**

**
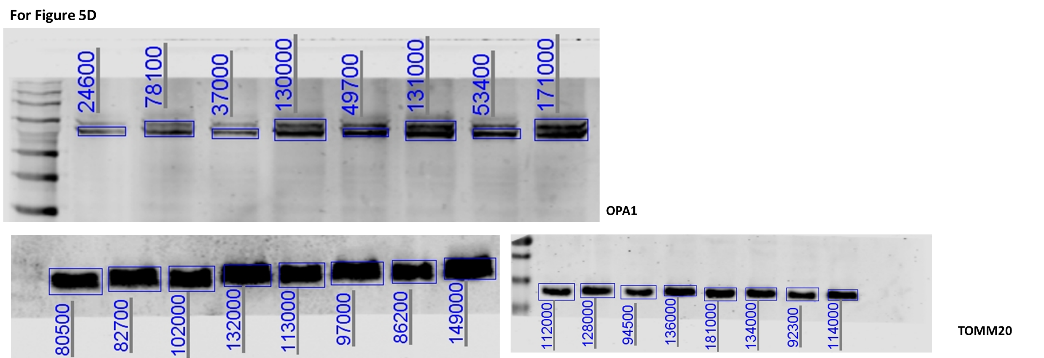
**

**
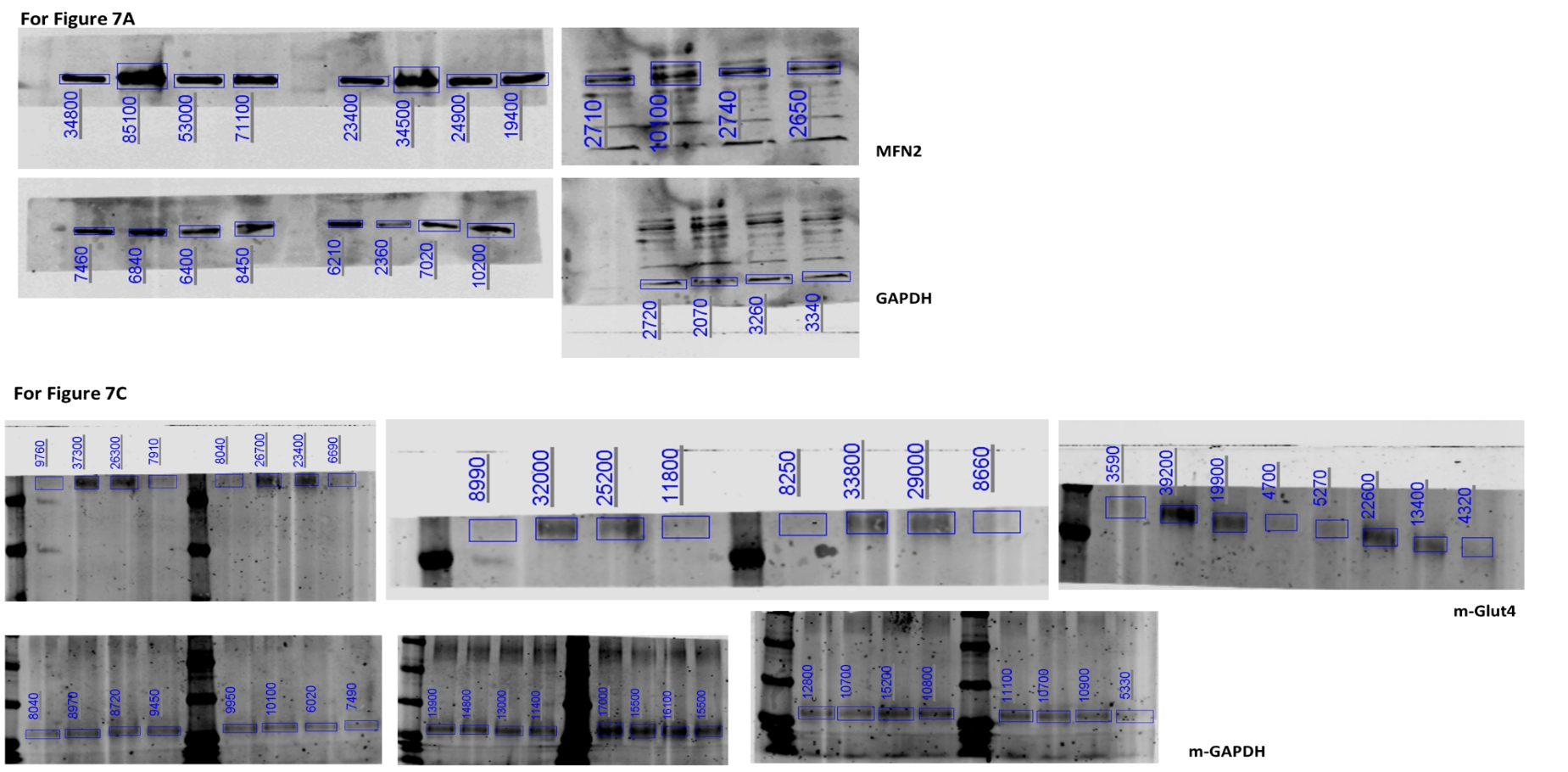
**

**
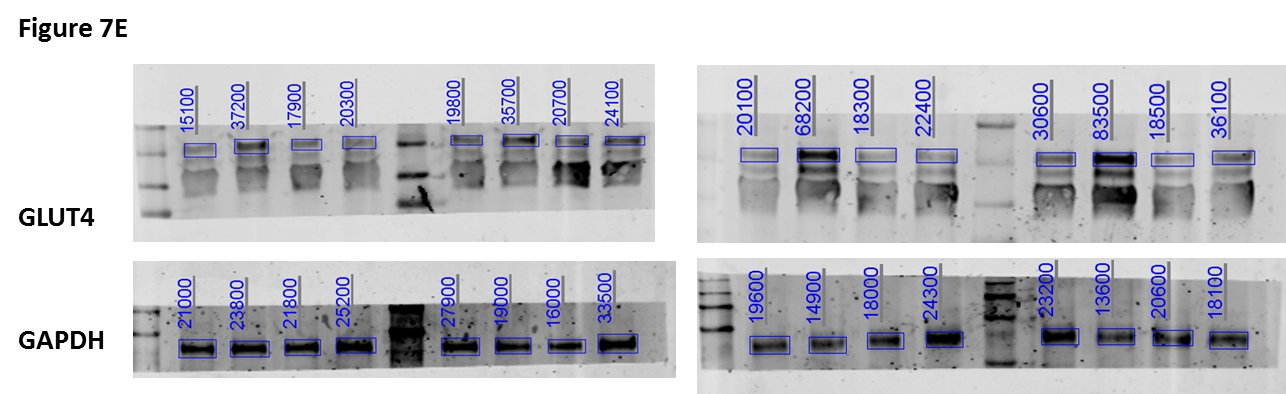
**

**WB1:** Immunoblots of U2OS cells with membrane photo overlay and band density for Figure 2A-E. Blue dashed boxes indicate the area used for the WB presented. Band density and intensity ratio (normalised to GAPDH) are included in the tables to the right.

**
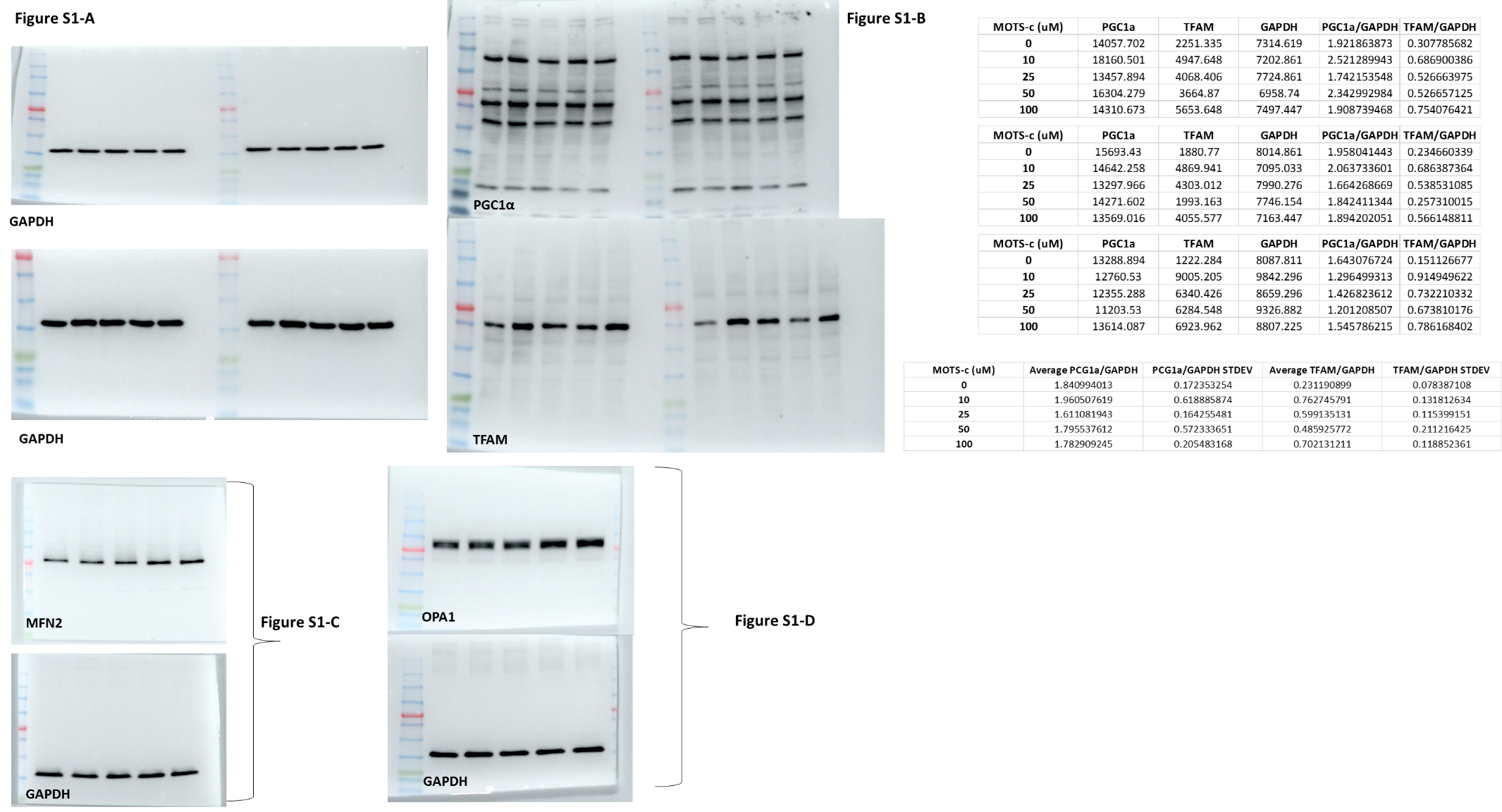
**

**WB2:** Immunoblots of 293T cells with membrane photo overlay and band density for Figure S1-2. Blue dashed boxes indicate the area used for the WB presented. Band density and intensity ratio (normalised to GAPDH) are included in the tables to the right.

**Figure S5**


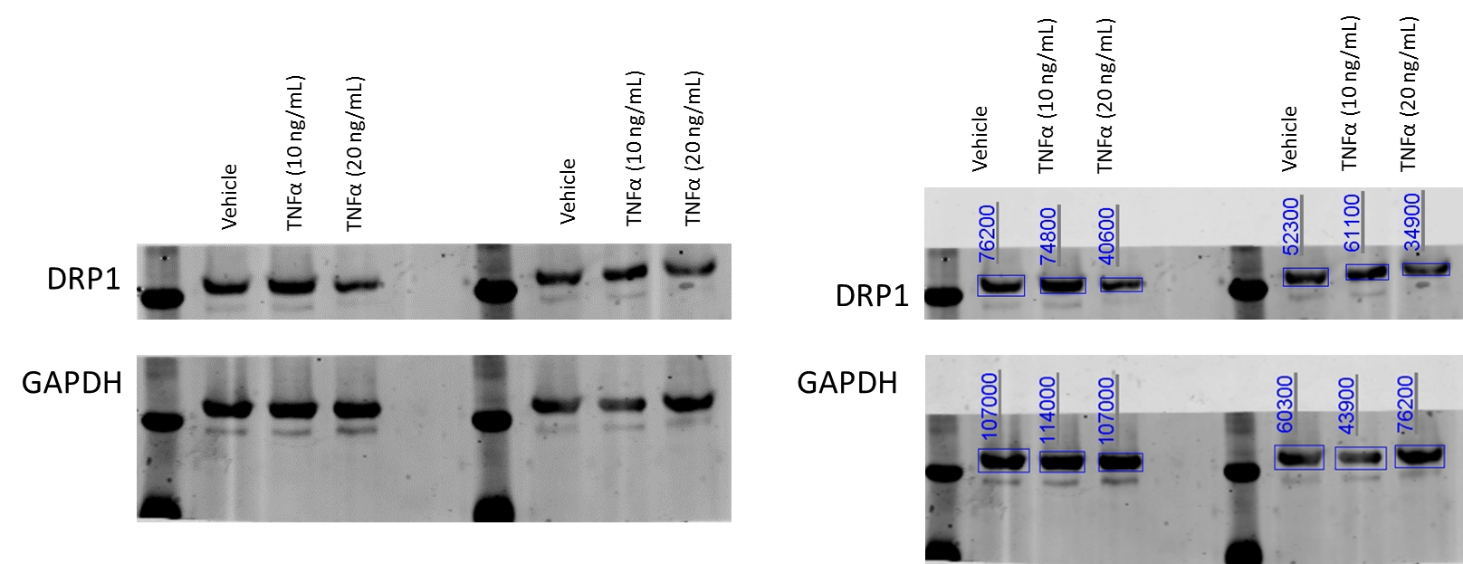

Supplement: Supplementary file 1 — Supplementary Information. [file 41598_2021_93735_MOESM1_ESM.docx]
